# Pharmacological Modulation of Perioperative Pain in Laparoscopic Bariatric Surgery: An Updated Evidence‐Based Narrative Review of Intravenous Lidocaine, Dexmedetomidine, Opioid‐Free and Opioid‐Sparing Anesthesia, and ERAS‐Based Protocols

**DOI:** 10.1155/anrp/6019290

**Published:** 2026-07-13

**Authors:** Carlos Zavaleta-Corvera, Erick Auccacusi Pachari, Julian Espinoza-Portilla, Wendy Farfán-Martinez, Mario Bolarte-Arteaga

**Affiliations:** ^1^ Faculty of Medicine, Universidad Cientifica del Sur, Lima, Lima, Peru, ucsur.edu.pe; ^2^ Teaching and Research Support Office (OADI), Daniel Alcides Carrión National Hospital, Callao, Lima, Peru; ^3^ Faculty of Medicine, Continental University, Lima, Lima, Peru

**Keywords:** bariatric anesthesia, dexmedetomidine, enhanced recovery after surgery, intravenous lidocaine, multimodal analgesia, opioid-free anesthesia, opioid-sparing anesthesia, postoperative nausea and vomiting, postoperative pain

## Abstract

Laparoscopic bariatric surgery is associated with specific analgesic challenges because opioid‐related adverse effects may be especially relevant in patients with obesity, obstructive sleep apnea, and cardiometabolic comorbidities. Inadequate perioperative analgesia in this population may impair early mobilization and functional recovery, while excessive opioid use increases the risk of respiratory depression, postoperative nausea and vomiting (PONV), and prolonged hospital stay. This narrative review synthesizes and critically appraises contemporary evidence on intravenous lidocaine, dexmedetomidine, opioid‐free or opioid‐sparing anesthesia, and enhanced recovery after surgery (ERAS)–based perioperative strategies in adult patients undergoing laparoscopic bariatric surgery. A structured literature search was conducted in PubMed/MEDLINE, Embase, Scopus, and the Cochrane Library from January 2018 to May 2026. A total of 41 studies were included, comprising randomized controlled trials, observational studies, pharmacokinetic investigations, systematic reviews, meta‐analyses, and ERAS implementation studies. Methodological quality was appraised using RoB 2.0, ROBINS‐I, and AMSTAR‐2 according to study design. Evidence for intravenous lidocaine remains heterogeneous and appears dependent on dosing strategy, infusion duration, and perioperative context; its antiemetic and anti‐inflammatory effects may be more consistent than its analgesic benefit when infusion is limited to the intraoperative period. Dexmedetomidine has been associated with reductions in perioperative opioid exposure and PONV through alpha‐2‐mediated sympatholysis and opioid‐sparing mechanisms, although effects on pain scores are variable and hemodynamic monitoring is required. Opioid‐free or opioid‐sparing strategies may reduce opioid exposure and PONV, but superiority over conventional anesthesia for pain control or length of stay is inconsistent, and protocol heterogeneity limits generalizability. ERAS pathways represent the most comprehensive and evidence‐supported framework for perioperative optimization, with benefit proportional to adherence and independent of any single pharmacologic agent. These pharmacologic strategies are best understood as mechanism‐based adjuncts to be selectively integrated within high‐adherence ERAS programs, not as universal standalone interventions. Rigorous multicenter randomized trials with standardized dosing, detailed ERAS adherence reporting, patient‐centered outcome measures, and subgroup analyses by procedure type, OSA status, and PONV risk are needed.

## 1. Introduction

Laparoscopic bariatric surgery is currently the most effective surgical treatment for morbid obesity and its associated metabolic comorbidities, with robust evidence supporting its superiority over nonsurgical interventions for long‐term weight loss and resolution of obesity‐related disease [1, 2]. However, perioperative analgesic management in this population presents distinct and clinically significant challenges that differ substantially from those encountered in standard elective surgery. Patients with morbid obesity exhibit altered pharmacokinetics, reduced functional residual capacity, and a high prevalence of obstructive sleep apnea (OSA), which collectively increase the risk of opioid‐related adverse events in the perioperative period [3, 4]. The combination of obesity‐related physiology, high‐risk comorbidities, and the specific demands of laparoscopic bariatric procedures creates an environment in which conventional opioid‐based analgesia carries elevated risk, and multimodal, opioid‐sparing strategies are not merely desirable but clinically necessary.

Several obesity‐specific physiological alterations directly influence perioperative analgesic requirements and outcomes. Obesity is a state of chronic low‐grade systemic inflammation characterized by elevated circulating concentrations of pro‐inflammatory cytokines, adipokines, and acute‐phase reactants, which amplify the surgical stress response and may heighten pain sensitivity [3]. Obesity‐related changes in respiratory mechanics, including reduced functional residual capacity, increased airway resistance, impaired diaphragmatic excursion, and a predisposition to perioperative atelectasis, render these patients particularly vulnerable to opioid‐induced respiratory depression and postoperative hypoxemia [4]. OSA is highly prevalent among patients presenting for bariatric surgery, with some estimates suggesting it may affect the majority of candidates depending on diagnostic criteria and screening strategy [5]; in this context, even modest opioid exposure may precipitate clinically relevant upper airway collapse and respiratory compromise in the early postoperative period [4, 5]. Beyond respiratory safety, postoperative nausea and vomiting (PONV) represents an additional high‐priority outcome in this population: The convergence of female predominance in bariatric cohorts, prolonged laparoscopic surgery, pneumoperitoneum, use of volatile anesthetics, and perioperative opioid administration creates a risk profile in which PONV may substantially delay oral intake, mobilization, and discharge [6]. Finally, undertreated postoperative pain carries its own functional consequences: Inadequate analgesia impairs early mobilization, delays return of gastrointestinal function, prolongs PACU and hospital length of stay, and reduces patient‐reported recovery quality [1]. The clinical objective in bariatric anesthesia is therefore not simply to minimize opioids, but to ensure that opioid reduction is achieved without compromising analgesia, respiratory safety, antiemetic control, or early functional recovery.

Multimodal analgesia has emerged as the operational strategy to meet this objective and should be understood as a mechanism‐based approach targeting multiple pain pathways simultaneously rather than merely accumulating pharmacologic agents [1, 7, 8]. Postoperative pain after laparoscopic bariatric surgery encompasses nociceptive, inflammatory, and neuropathic or hyperalgesic components: Peripheral sensitization driven by surgical tissue injury, cytokine release, and prostaglandin activation amplifies nociceptive input from the operative site, while central sensitization, including wind‐up and spinal hyperexcitability, contributes to postoperative allodynia and heightened analgesic requirements [8]. Multimodal analgesia achieves opioid‐sparing by simultaneously intervening across multiple pathways: voltage‐gated sodium channels (lidocaine), inflammatory and prostaglandin cascades (nonsteroidal anti‐inflammatory drugs and acetaminophen), N‐methyl‐D‐aspartate receptors (ketamine and magnesium), alpha‐2 adrenergic pathways (dexmedetomidine), and peripheral afferent transmission (regional anesthesia and transversus abdominis plane blocks) [1, 7, 8]. When combined together, synergistic or additive effects allow adequate analgesia at lower individual doses, reducing both opioid consumption and the adverse event burden associated with any single high‐dose agent. This conceptual framework also clarifies that opioid‐free anesthesia (OFA) and opioid‐sparing anesthesia are not equivalent: OFA aims to eliminate opioids entirely from the intraoperative and immediate postoperative period, relying exclusively on nonopioid combinations, whereas opioid‐sparing strategies minimize opioid use within a multimodal framework without mandating complete elimination.

Among systemic pharmacologic agents, intravenous lidocaine and dexmedetomidine have attracted particular interest because of their mechanistic profiles, which align well with the analgesic and physiological demands of bariatric surgery. Intravenous lidocaine has been evaluated in this population because of its sodium channel blocking, antihyperalgesic, anti‐inflammatory, and pharmacokinetically complex profile in patients with obesity: Meta‐analytic evidence supports reductions in opioid consumption and PONV, particularly with extended infusion strategies, while pharmacokinetic investigations confirm that obesity substantially alters lidocaine distribution and clearance [9–12]. Dexmedetomidine, a highly selective alpha‐2 adrenergic agonist, has been investigated for its opioid‐sparing and antiemetic effects through sympatholysis and dorsal horn inhibition, with its respiratory‐sparing sedative profile providing a mechanistic rationale for use in OSA‐predominant bariatric cohorts; consistent benefits for perioperative opioid reduction and PONV have been reported across multiple meta‐analyses and randomized trials, particularly when administered as a continuous infusion rather than as a single bolus [13–17]. Both agents are increasingly incorporated into enhanced recovery after surgery (ERAS) pathways, which aim to optimize perioperative outcomes through standardized, evidence‐based care processes [1].

The growing interest in OFA in bariatric surgery reflects the intuitive appeal of eliminating opioids in a high‐risk population. However, OFA is not a single intervention but a family of heterogeneous protocols that may combine dexmedetomidine, lidocaine, ketamine, magnesium, dexamethasone, acetaminophen, nonsteroidal anti‐inflammatory drugs, and regional techniques in differing doses, sequences, and combinations [18, 19]. This heterogeneity has important implications for interpreting the evidence: benefits observed in one OFA protocol may not be attributable to the opioid‐free concept itself, but to specific components within that protocol. Recent evidence suggests that OFA may reduce PONV and postoperative opioid exposure, but its effects on pain scores and length of stay are inconsistent, partly because protocols vary widely in pharmacologic composition and rescue analgesia thresholds [18–21]. Furthermore, OFA does not guarantee that patients will not require opioid rescue analgesia in the postoperative period, and protocols that avoid opioids completely at the cost of inadequate analgesia carry their own risks [18, 19]. Opioid‐sparing anesthesia, which rationalizes and minimizes opioid use within a multimodal framework rather than eliminating it entirely, may represent a more pragmatic and clinically acceptable standard for most bariatric patients [18, 21].

ERAS programs provide the organizational framework within which pharmacologic adjuncts are most meaningfully evaluated. ERAS is not a pharmacologic intervention but a structured, multidisciplinary perioperative care pathway that integrates preoperative patient education and carbohydrate loading, fluid optimization, PONV prophylaxis, multimodal analgesia, early feeding, early mobilization, and defined discharge criteria [1]. Adherence to ERAS Society recommendations has been associated with shorter hospital stay without an increase in complications or readmissions in bariatric surgery, with a clear dose–response relationship between adherence level and outcome benefit [22]. Large implementation studies further confirm that ERAS adoption reduces LOS, complications, PONV, and direct costs when implemented with sufficient protocol fidelity [2, 23–29]. Critically, the analgesic benefit of individual agents, such as lidocaine or dexmedetomidine, may be substantially modified by background ERAS implementation: ERAS adherence is not merely a confounder but an effect modifier, and pharmacologic adjuncts should be evaluated and implemented as components of structured ERAS programs rather than as isolated interventions [1, 22, 23].

Although several multimodal analgesic interventions have been evaluated in bariatric surgery, including ketamine, magnesium, nonsteroidal anti‐inflammatory drugs, gabapentinoids, and regional anesthesia techniques, such as TAP blocks, whose procedure‐specific role is addressed in dedicated evidence‐based recommendations [1, 7], the present review focuses on intravenous lidocaine, dexmedetomidine, opioid‐free or opioid‐sparing anesthesia, and ERAS pathways because these strategies represent systemic pharmacologic and protocol‐based approaches most frequently incorporated into contemporary institutional bariatric anesthesia protocols and most extensively addressed in the recent literature. Regional techniques and other adjuncts are acknowledged as relevant components of multimodal analgesia and are referenced where they intersect with the primary domains, but were not selected as primary review domains in order to maintain a focused synthesis on systemic intravenous pharmacologic modulation and its integration into ERAS‐based perioperative care.

Existing reviews have evaluated individual agents or ERAS pathways in isolation, but evidence published between 2024 and 2026 has substantially expanded the literature on intravenous lidocaine, dexmedetomidine, OFA, and ERAS implementation in bariatric surgery [10, 13, 14, 18–20, 23, 30–34]. An updated synthesis is therefore needed to clarify how these interventions should be interpreted mechanistically and clinically within contemporary ERAS‐based bariatric care. This narrative review aims to synthesize and critically appraise contemporary evidence on intravenous lidocaine, dexmedetomidine, opioid‐free or opioid‐sparing anesthesia, and ERAS‐based multimodal perioperative strategies in adult patients undergoing laparoscopic bariatric surgery, with emphasis on postoperative pain, opioid consumption, PONV, recovery outcomes, safety, and clinical implementation.

## 2. Methods

### 2.1. Design

This work was designed as an evidence‐based narrative review. A narrative approach was selected because the available literature is heterogeneous in study design, interventions, dosing regimens, comparators, and outcome definitions, making formal meta‐analytic pooling methodologically inappropriate. The review was conducted following principles of methodological rigor and transparency recommended for integrative narrative syntheses, with particular attention to differentiating findings by study design and level of evidence, and to distinguishing statistically significant results from clinically meaningful effects.

Although a structured search strategy, predefined eligibility criteria, dual‐reviewer screening, and design‐specific methodological appraisal were used to improve transparency and reproducibility, this article was not designed as a formal systematic review. No prospective protocol was registered, gray literature and trial registries were not systematically searched, and no quantitative synthesis, meta‐analysis, or formal certainty‐of‐evidence grading was performed. The purpose of the structured approach was to support a rigorous evidence‐based narrative synthesis, not to generate pooled effect estimates or procedure‐specific recommendations equivalent to those produced by systematic reviews or guideline panels.

### 2.2. Search Strategy

A structured literature search was conducted in PubMed/MEDLINE, Embase, Scopus, and the Cochrane Library. The search covered publications from January 2018 to May 2026. The search was performed independently for each of the four intervention domains using separate strategies combining Medical Subject Headings (MeSH) terms and free‐text equivalents. Domain 1 (intravenous lidocaine) combined terms including bariatric surgery, laparoscopic sleeve gastrectomy, Roux‐en‐Y gastric bypass, lidocaine, intravenous lidocaine, lidocaine infusion, postoperative pain, opioid consumption, PONV, quality of recovery, and length of stay. Domain 2 (dexmedetomidine) combined the following: bariatric surgery, dexmedetomidine, alpha‐2 agonist, opioid‐sparing, PONV, PACU, bradycardia, and hypotension. Domain 3 (OFA/opioid‐sparing) combined the following: OFA, opioid‐sparing anesthesia, multimodal anesthesia, bariatric surgery, and recovery outcomes. Domain 4 (ERAS) combined the following: ERAS, bariatric surgery, adherence, implementation, length of stay, complications, readmissions, and cost. Reference lists of included articles and recent systematic reviews were also screened for additional eligible studies.

### 2.3. Eligibility Criteria

Studies were eligible if they enrolled adult patients (aged 18 years or older) undergoing laparoscopic bariatric surgery. The intervention must have involved intravenous lidocaine, dexmedetomidine, opioid‐free or opioid‐sparing anesthetic regimens, or structured ERAS pathways in the perioperative period. Eligible study designs included RCTs, prospective and retrospective observational studies, pharmacokinetic studies, systematic reviews, and meta‐analyses. Only studies published in English or Spanish involving human subjects were considered. Studies were excluded if they were case reports, narrative commentaries without primary data, letters, editorials, conference abstracts without full text, animal research, pediatric studies, or if the surgical procedure was not performed laparoscopically. Gray literature, preprints, and trial registries were not systematically searched; where referenced, they were used for contextual purposes only.

### 2.4. Study Selection and Data Extraction

**FIGURE 1:**
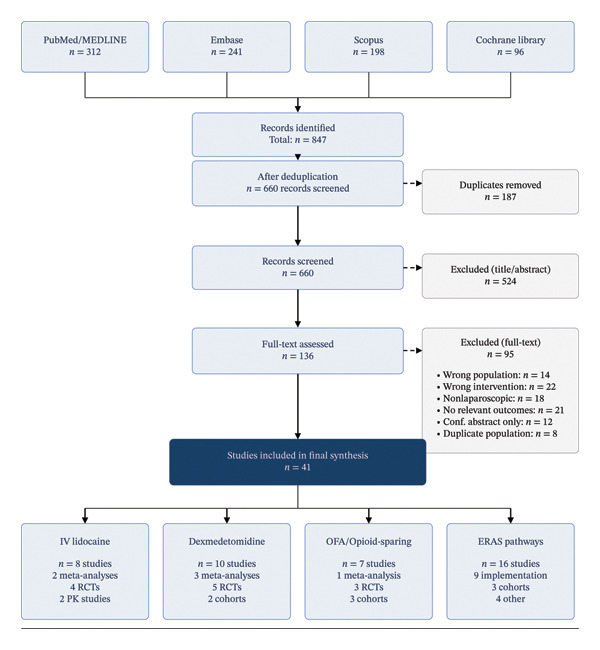
Structured study selection flow diagram. Flow data: Records identified: PubMed/MEDLINE *n* = 312, Embase *n* = 241, Scopus *n* = 198, Cochrane Library *n* = 96 (Total: 847). After removal of 187 duplicates: 660 screened. Records excluded at title/abstract: 524. Full‐text articles assessed: 136. Full‐text excluded: 95 (wrong population: 14; wrong intervention/comparator: 22; nonlaparoscopic/nonbariatric: 18; no relevant outcomes: 21; conference abstract only: 12; duplicate/overlapping population: 8). Studies included in final synthesis: 41. Abbreviations: MA = meta‐analysis; RCT = randomized controlled trial; PK = pharmacokinetic; OFA = opioid‐free anesthesia; ERAS = enhanced recovery after surgery. Dashed arrows = excluded records. Domain totals overlap (some studies inform multiple domains).

Titles and abstracts were screened for eligibility, followed by full‐text assessment of potentially eligible records. Two reviewers (C.Z.‐C. and E.A.P.) performed screening and data extraction independently; disagreements were resolved through discussion and, when necessary, consensus with a third reviewer (J.E.P.). Information extracted from each study included the following: study design, country, sample size, type of bariatric procedure, intervention and comparator, dosing regimen and administration strategy, background ERAS adherence, primary and secondary outcomes, adverse events, and methodological limitations. The selection process is summarized in Figure 1.

### 2.5. Methodological Appraisal

Methodological quality was appraised according to study design. RCTs were assessed using the Cochrane Risk of Bias 2.0 tool (RoB 2.0). Observational studies were evaluated using the relevant domains of the ROBINS‐I framework. Systematic reviews and meta‐analyses were appraised using AMSTAR‐2. Pharmacokinetic studies were evaluated through narrative assessment of methodological limitations including sample size, dosing strategy, measurement of serum concentrations, and representativeness of the study population. Because this was a narrative review, quality judgments were not used to exclude studies but were incorporated when interpreting the certainty and applicability of findings.

### 2.6. Outcome Framework

Outcomes were evaluated across multiple dimensions reflecting the multidimensional nature of perioperative recovery. Pain scores, while commonly reported, were considered necessary but insufficient as standalone measures, since numerical pain ratings may not capture the functional impact of analgesia. Opioid consumption was used as a marker of opioid‐sparing rather than as a direct measure of analgesic quality, recognizing that lower opioid use does not automatically imply superior pain control. PONV was considered a high‐priority outcome in bariatric surgery given its specific risk burden and its effect on oral intake, mobilization, patient satisfaction, and time to discharge. PACU length of stay was distinguished from hospital length of stay, recognizing that interventions reducing PACU time may not translate to shorter total hospitalization without broader ERAS implementation. Readmissions, complications, and direct costs were evaluated as safety and resource outcomes. Patient‐reported measures, including Quality of Recovery‐15 (QoR‐15) and overall patient satisfaction, were prioritized where available as endpoints most proximal to the patient experience.

### 2.7. Evidence Synthesis

The evidence is presented thematically across four domains: intravenous lidocaine studies and meta‐analyses, dexmedetomidine‐based perioperative strategies, opioid‐free or opioid‐sparing anesthesia protocols, and ERAS implementation and adherence studies. Within each domain, findings were interpreted according to study design, prioritizing evidence from systematic reviews and meta‐analyses, followed by RCTs, prospective observational studies, retrospective cohorts, pharmacokinetic investigations, and implementation studies. For each domain, a brief mechanistic rationale precedes the evidence summary to provide the conceptual basis for interpreting clinical findings. Study‐level heterogeneity in dosing, comparators, and outcome definitions is acknowledged throughout. ERAS background adherence was considered a potential effect modifier when interpreting pharmacologic intervention results.

### 2.8. Protocol Registration and Ethics

This review was not registered in PROSPERO because narrative reviews are not eligible for registration. As this study involved secondary analysis of previously published data, institutional ethics committee approval was not required.

## 3. Results

### 3.1. Study Selection and Included Evidence

The search identified 847 records across the four databases (PubMed/MEDLINE: 312; Embase: 241; Scopus: 198; and Cochrane Library: 96). After removal of 187 duplicates, 660 records were screened by title and abstract. Following exclusion of 524 records, 136 full‐text articles were assessed. Of these, 95 were excluded for the following reasons: wrong population (*n* = 14), wrong intervention or comparator (*n* = 22), nonlaparoscopic or nonbariatric procedure (*n* = 18), no relevant perioperative outcomes (*n* = 21), conference abstract without full text (*n* = 12), and overlapping or duplicate populations (*n* = 8). A total of 41 studies were included in the final narrative synthesis, comprising 9 systematic reviews or meta‐analyses, 14 RCTs, 9 retrospective observational studies, 3 pharmacokinetic studies, and 6 ERAS implementation studies. The selection process is summarized in Figure 1.

Overall methodological quality varied considerably. Among RCTs, the most common sources of concern were deviations from intended interventions, small sample sizes, and incomplete reporting of allocation concealment or blinding procedures. Observational studies were primarily limited by confounding by indication and variability in background ERAS adherence. Meta‐analyses were informative but differed in eligibility criteria, outcome definitions, and approaches to heterogeneity. Pharmacokinetic studies were limited by modest sample sizes and limited demographic representativeness.

### 3.2. Intravenous Lidocaine in Laparoscopic Bariatric Surgery

#### 3.2.1. Mechanistic Rationale

Intravenous lidocaine exerts its perioperative effects through at least three partially independent mechanisms. First, as a voltage‐gated sodium channel blocker, it reduces peripheral and central nociceptive transmission by attenuating ectopic neuronal discharge and dampening dorsal horn excitability. Second, through anti‐inflammatory activity including inhibition of leukocyte degranulation, reduction of pro‐inflammatory cytokine release (notably interleukin‐6 and tumor necrosis factor‐alpha), and modulation of eicosanoid signaling, it attenuates the systemic inflammatory response to surgical injury. Third, at supra‐analgesic concentrations achieved with extended infusion, lidocaine may exert antihyperalgesic effects by suppressing central sensitization pathways. In the context of laparoscopic bariatric surgery, where visceral distension and pneumoperitoneum contribute to a distinct pain phenotype, lidocaine may also attenuate enteric nervous system reactivity and accelerate postoperative gastrointestinal recovery. Critically, the pharmacokinetics of lidocaine are substantially altered by obesity: Increased adipose distribution volume, altered protein binding, modified hepatic blood flow, and potentially reduced clearance increase the risk of drug accumulation, especially during extended infusions. Adjusted body‐weight dosing has therefore been proposed as a safer dosing strategy in this population. Toxicity risk, including central nervous system symptoms (perioral numbness, dizziness, and seizures) and cardiovascular effects (QRS widening, arrhythmias, and hypotension), underscores the need for protocolized monitoring.

#### 3.2.2. Evidence Summary

Table 1 summarizes the main studies. The most comprehensive meta‐analytic evidence derives from Hung et al. [9], who reported that intraoperative intravenous lidocaine was associated with reduced 24‐h equivalent morphine consumption (MD: −11.97 mg; 95% CI: −23.12 to −0.83), lower early postoperative pain scores at 1–3 h (MD: −0.77; 95% CI: −1.50 to −0.04), and shorter hospital length of stay (MD: −8.93 h; 95% CI: −13.41 to −4.44). However, sensitivity analyses demonstrated that the evidence supporting reductions in opioid consumption, pain scores, and length of stay was weak, whereas more consistent benefit was observed for time to first opioid requirement and quality‐of‐recovery outcomes. A more recent meta‐analysis by Barbosa et al. [10] confirmed reductions in intraoperative and postoperative opioid requirements and PONV while identifying important heterogeneity linked to infusion duration and dosing strategy (AMSTAR‐2: moderate confidence for both).

**TABLE 1:** Key studies evaluating intravenous lidocaine in laparoscopic bariatric surgery.

Study	Design	*N*	Procedure	Lidocaine regimen (dose/duration)	Comparator	ERAS background	Methodological appraisal	Key finding
Hung et al. [9]	Meta‐analysis (8 RCTs)	523	Mixed bariatric laparoscopic	Intraoperative IV infusion (variable)	Placebo/saline	Not reported	AMSTAR‐2: Moderate confidence. Heterogeneity by infusion duration.	35% opioid reduction; reduced pain, PONV, LOS; no increase in adverse events
Barbosa et al. [10]	Systematic review/meta‐analysis	7 RCTs/640	Laparoscopic bariatric surgery	Pre‐/intraoperative IV lidocaine; dose varies by trial	Saline placebo	Recovery quality focus; ERAS not specified	RCT‐only meta‐analysis; useful synthesis but regimen heterogeneity	Improved recovery score, reduced morphine use, PONV, and LOS; no bowel recovery difference
Sakata et al. [35]	RCT (double‐blind)	58	Laparoscopic bariatric surgery	IV lidocaine 1.5 mg/kg before induction and then 2 mg/kg/h intraop	Saline placebo	Not specified	Small RCT; focused on GI recovery/discharge and opioid use	Reduced 24‐h morphine and sevoflurane use, but no difference in flatus, discharge criteria, or side effects
Plass et al. [36]	RCT (prospective, blinded)	176	Laparoscopic bariatric surgery	IV lidocaine 1.5 mg/kg bolus, then 2 mg/kg/h intraop, and then 1 mg/kg/h for 1 h in PACU	Saline placebo	Enhanced recovery protocol	Well‐designed RCT; effect statistically significant but clinically small	Slightly reduced 3‐day oxycodone use, but no meaningful differences in pain, PONV, bowel recovery, or LOS
de Oliveira et al. [37]	RCT	60	Laparoscopic gastroplasty/gastric bypass	IV lidocaine 1.5 mg/kg before induction and then 2 mg/kg/h intraoperatively	Saline placebo	Not specified	Small RCT; assessed pain, morphine use, IL‐6, nausea	Reduced early pain, 24‐h morphine use, nausea, and delayed first morphine request; no IL‐6 reduction
Duarte‐Medrano et al. [30]	Retrospective cohort	50	Laparoscopic bariatric surgery	Intraoperative IV lidocaine infusion; dose not specified in abstract	Standard care	Partial ERAS	Small retrospective study; limited regimen details	Lower pain at 1 h and trend toward less nausea/PONV with lidocaine
Zhang et al. [11]	Prospective PK clinical study	58	Obese: Laparoscopic sleeve gastrectomy; Controls: Laparoscopic cholecystectomy	IV lidocaine 1.5 mg/kg bolus; obese by ABW, controls by TBW	Normal‐weight patients	Not specified	Comparative PK/safety study, not efficacy trial	Obesity altered lidocaine PK, but levels stayed < 5 μg/mL and no serious lidocaine‐related AEs occurred
Carabalona et al. [12]	Prospective clinical trial	42	Laparoscopic bariatric surgery	IV lidocaine: 1.5 mg/kg bolus and then 2 mg/kg/h; reduced to 1 mg/kg/h after skin closure until PACU discharge	None	Not specified	Single‐arm pharmacokinetic safety study	Serum lidocaine remained within accepted range: 1.5–5 μg/mL

*Note:* IL‐6 = interleukin‐6, PK = pharmacokinetic, RoB 2.0 = Cochrane risk of bias 2.0, AMSTAR‐2 = Assessing the Methodological Quality of Systematic Reviews 2, ROBINS‐I = Risk Of Bias In Non‐randomized Studies of Interventions, NS = not specified in accessible publication.

Abbreviations: ABW = adjusted body weight, LOS = length of stay, PONV = postoperative nausea and vomiting, RCT = randomized controlled trial.

Among individual RCTs, Sakata et al., Plass et al., and de Oliveira et al. used broadly similar lidocaine dosing strategies, typically a 1.5 mg/kg bolus followed by a 2 mg/kg/h intraoperative infusion, but differed substantially in infusion duration, background analgesic co‐interventions, postoperative rescue protocols, and selected endpoints [35–37]. Sakata et al. administered lidocaine until the end of surgery in patients undergoing laparoscopic gastric bypass and reported reduced 24‐h morphine consumption and lower sevoflurane requirements, without significant differences in time to first flatus, adverse effects, or time to meet discharge criteria. De Oliveira et al. used a comparable intraoperative infusion strategy until skin closure and found lower 24‐h morphine consumption, delayed time to first morphine requirement, lower early postoperative pain scores, and reduced nausea at 24 h, without differences in IL‐6 concentrations. By contrast, Plass et al. extended lidocaine into the recovery period for one additional hour within a broader multimodal regimen that included ketamine, paracetamol, nefopam, parecoxib, and wound infiltration; this study demonstrated a statistically significant but modest reduction in postoperative oxycodone consumption, without clear improvement in pain scores, PONV, bowel recovery, or hospital length of stay. Together, these trials suggest that the clinical effect of intravenous lidocaine in bariatric surgery is not uniform and may depend less on the presence of lidocaine alone than on infusion duration, background multimodal analgesia, rescue opioid thresholds, and the outcome selected for assessment. A retrospective cohort by Duarte‐Medrano et al. [30] found a limited analgesic effect but a reduction in PONV incidence.

Pharmacokinetic data from Zhang et al. [11] confirmed that adjusted body‐weight dosing achieves safe plasma concentrations. Carabalona et al. [12] emphasized the need for monitoring during extended infusions. Collectively, the evidence suggests that the clinical benefit of intravenous lidocaine is highly dependent on infusion duration, dose, and perioperative context.

### 3.3. Dexmedetomidine in Laparoscopic Bariatric Surgery

#### 3.3.1. Mechanistic Rationale

Dexmedetomidine is a highly selective alpha‐2 adrenergic agonist (alpha‐2:alpha‐1 selectivity ratio approximately 1600:1) that reduces sympathetic nervous system tone through presynaptic inhibition in the locus coeruleus, spinal cord, and peripheral sympathetic terminals. Its analgesic mechanism involves activation of alpha‐2 receptors in the dorsal horn and in supraspinal descending inhibitory pathways, which reduce nociceptive transmission without producing respiratory depression at clinical doses. This respiratory‐sparing quality is particularly relevant in OSA patients. Opioid‐sparing is achieved both through direct analgesia and through attenuation of the hemodynamic and sympathetic stress responses to surgical stimulation. The antiemetic benefit is largely indirect through reduced perioperative opioid exposure, with a possible direct central antiemetic effect through inhibition of the nucleus tractus solitarius and area postrema. The pharmacokinetic rationale for continuous infusion over single bolus relates to the need for sustained alpha‐2 receptor occupancy; bolus administration achieves transient peak concentrations that may produce hemodynamic instability without sustaining the receptor‐level effects associated with benefit. Bradycardia and hypotension are dose‐dependent effects resulting from reduced sympathetic output and increased vagal tone.

#### 3.3.2. Evidence Summary

Table 2 summarizes the key studies. Altamimi et al. [13] pooled six RCTs (485 participants) and reported a significant reduction in intraoperative fentanyl consumption and PONV, with mixed results for pain scores. Subramaniam et al. [14] confirmed a consistent and clinically meaningful antiemetic effect across 13 RCTs. Verret et al. [31], in a broad Bayesian meta‐analysis of surgical patients receiving general anesthesia, evaluated the effect of dexmedetomidine on patient‐centered postoperative outcomes, providing supportive but indirect evidence for its role as an opioid‐minimizing perioperative adjunct.

**TABLE 2:** Key studies evaluating dexmedetomidine in laparoscopic bariatric surgery.

Study	Design	Procedure	Dexmedetomidine regimen	Comparator	Pain outcome	PONV/opioid effect	Methodological appraisal/key finding
Altamimi et al. [13]	Meta‐analysis (6 RCTs)	Laparoscopic bariatric	Various infusion protocols	Opioid‐based	Mixed pain results	Reduced fentanyl and PONV	AMSTAR‐2: Moderate confidence. Consistent opioid/PONV benefit; heterogeneous pain results
Subramaniam et al. [14]	Meta‐analysis (13 RCTs)	Laparoscopic bariatric	Perioperative infusion	Various	Not primary	Consistent PONV reduction	AMSTAR‐2: Moderate confidence. Strongest evidence for antiemetic effect
Verret et al. [31]	Systematic review and Bayesian meta‐analysis	Mixed surgical populations under general anesthesia	Perioperative dexmedetomidine	Placebo, standard care, or various comparators	Patient‐centered postoperative outcomes assessed	Supportive evidence for opioid‐minimizing perioperative use, but not bariatric‐specific	AMSTAR‐2: Moderate confidence. Broad surgical evidence with indirect applicability to laparoscopic bariatric surgery
Nam et al. [15]	Retrospective cohort	Laparoscopic bariatric	Intraoperative infusion	Remifentanil	Pain reduction	Less PONV and opioids	ROBINS‐I: Moderate risk (selection bias, confounding by indication)
Zeeni et al. [16]	Double‐blind RCT	Laparoscopic bariatric	Dexmedetomidine infusion	Morphine‐based	No significant difference at 24 h	48% total opioid reduction; less PONV	RoB 2.0: Some concerns (incomplete blinding). Total opioid reduction despite null pain result
Narejo et al. [38]	Pilot clinical study	Laparoscopic sleeve gastrectomy	Dexmedetomidine‐based	Remifentanil	Better pain control	Improved recovery profile	RoB 2.0: High risk (small sample, limited allocation reporting)
Ranganathan et al. [17]	RCT	Roux‐en‐Y gastric bypass	Intraoperative bolus	Placebo	No significant benefit	No reduction in opioid use	RoB 2.0: Low risk. Key null finding; consistent with bolus vs infusion pharmacological rationale
Nair et al. [32]	Systematic review and meta‐analysis	Bariatric and metabolic surgery	Dexmedetomidine‐based strategies	Remifentanil‐based strategies	Analgesic efficacy synthesized across RCTs	Comparative evidence on dexmedetomidine versus remifentanil; indirect support for opioid‐sparing bariatric anesthesia	AMSTAR‐2: Moderate confidence. Evidence limited by heterogeneity among included RCTs
Sprung et al. [39]	Retrospective observational	Laparoscopic bariatric	Nonopioid strategy incl. dexmedetomidine	Opioid‐based	Not reported	Less rescue antiemetics in PACU	ROBINS‐I: Moderate risk (selection bias)

*Note:* Methodological appraisal tools as in Table 1. PONV = postoperative nausea and vomiting.

Abbreviations: PACU = postanesthesia care unit, RCT = randomized controlled trial.

Among individual studies, Nam et al. [15] found in a retrospective cohort of 342 patients that intraoperative dexmedetomidine was associated with significantly lower PONV incidence and reduced postoperative opioid requirements versus remifentanil. Zeeni et al. [16] demonstrated nearly 50% total perioperative opioid reduction and lower PONV rates, although morphine at 24 h did not differ significantly. Narejo et al. [38] reported improved postoperative pain control, decreased rescue opioid use, and a better ERAS‐aligned recovery profile following sleeve gastrectomy when dexmedetomidine was incorporated into the anesthetic strategy. However, procedure‐specific recommendations remain more cautious. Unlike ERAS Society guidance for bariatric surgery, the PROSPECT guidelines for laparoscopic sleeve gastrectomy do not currently recommend routine use of either dexmedetomidine or intravenous lidocaine because of inconsistent procedure‐specific evidence and uncertainty regarding the balance between clinical benefit and adverse effects.

An important null finding comes from Ranganathan et al. [17], in which intraoperative dexmedetomidine bolus did not reduce postoperative pain or narcotic use, consistent with the pharmacological rationale that bolus administration may be insufficient to sustain opioid‐sparing effects. Nair et al. [32] synthesized randomized evidence comparing remifentanil and dexmedetomidine in bariatric and metabolic surgery, supporting the need to balance opioid‐sparing benefits with hemodynamic considerations. Sprung et al. [39] found reduced rescue antiemetic use in the PACU with nonopioid‐leaning strategies incorporating dexmedetomidine.

### 3.4. Opioid‐Free and Opioid‐Sparing Anesthesia

#### 3.4.1. Conceptual Framework

OFA and opioid‐sparing anesthesia represent a spectrum rather than a binary classification. OFA aims to eliminate intraoperative opioids entirely and minimize or avoid postoperative opioid rescue, relying on combinations of nonopioid agents. Opioid‐sparing strategies minimize opioid use within a multimodal framework without mandating complete elimination. The critical distinction is that OFA is not a single protocol but a heterogeneous family of approaches combining dexmedetomidine, lidocaine, ketamine, magnesium, dexamethasone, acetaminophen, nonsteroidal anti‐inflammatory drugs, and regional techniques in widely varying doses, sequences, and rationales. This protocol heterogeneity creates an inherent interpretive challenge. Furthermore, the assumption that eliminating intraoperative opioids will automatically reduce postoperative opioid requirements is not universally supported. Protocols that avoid opioids completely at the cost of inadequate analgesia carry the risk of hemodynamic instability, awareness, and patient dissatisfaction. Opioid‐sparing multimodal care may represent a more clinically acceptable standard for the majority of bariatric patients.

#### 3.4.2. Evidence Summary

Table 3 summarizes the evidence. Bersot et al. [18], in a broader meta‐analysis of opioid‐free versus opioid‐based anesthesia in laparoscopic surgery, pooled 16 RCTs and found reduced PONV and opioid consumption, although the indirectness of this evidence should be considered when extrapolating to bariatric surgery. This aligns with the mechanistic expectation that OFA primarily benefits PONV and opioid exposure through reduction of perioperative opioid triggers rather than through superior analgesia.

**TABLE 3:** Key studies evaluating opioid‐free and opioid‐sparing anesthesia in laparoscopic bariatric surgery.

Study	Design	*N*	OFA/opioid‐sparing protocol (main components)	Comparator	Pain outcome	PONV/opioid effect	LOS/other	Methodological appraisal/key finding
Bersot et al. [18]	Meta‐analysis (16 RCTs)	1322	OFA (variable components: dexmedetomidine, lidocaine, ketamine, magnesium, NSAIDs)	Conventional anesthesia	No consistent pain benefit	Reduced PONV and opioid consumption	No consistent LOS benefit	AMSTAR‐2: Moderate confidence. Largest OFA meta‐analysis; benefit driven by antiemetic components rather than opioid elimination per se
Olausson et al. [20]	Multicenter RCT	NS	Dexmedetomidine + lidocaine + ketamine + dexamethasone	Conventional opioid‐based	No significant difference at 24 h	Less PACU opioid rescue; less PONV	Not reported	RoB 2.0: Low risk. Most rigorous individual OFA trial. Null pain result despite PONV benefit
Dagher et al. [33]	Prospective study	NS	Opioid‐sparing multimodal protocol	Standard care	No LOS difference	Lower morphine; higher patient satisfaction	No LOS difference	ROBINS‐I: Moderate risk. Patient satisfaction endpoint adds patient‐centered dimension
Mieszczanski et al. [19]	Narrative review	N/A	OFA (multiple heterogeneous protocols)	N/A	Inconsistent across trials	PONV benefits most consistent	Not pooled	Highlights protocol heterogeneity as main evidence limitation; OSA rationale discussed
Silva et al. [40]	Retrospective comparative	NS	Lidocaine + dexmedetomidine + magnesium + methadone	Opioid‐based strategies	90% reduction in moderate–severe PACU pain	Improved	Shorter PACU stay	ROBINS‐I: Moderate risk (selection bias, retrospective design). Extreme effect size warrants cautious interpretation
Perez et al. [21]	Prospective single‐blind RCT	NS	Dexmedetomidine + lidocaine + ketamine (fully OFA)	Conventional anesthesia	No significant difference	No reduction in opioid use or PONV	No LOS reduction	RoB 2.0: Some concerns (blinding). Important null finding constraining universal OFA claims

*Note:* NSAID = nonsteroidal anti‐inflammatory drug, PONV = postoperative nausea and vomiting. Methodological appraisal tools as in Table 1.

Abbreviations: LOS = length of stay, OFA = opioid‐free anesthesia, PACU = postanesthesia care unit, RCT = randomized controlled trial.

Olausson et al. [20] in a multicenter RCT found reductions in PACU opioid rescue and PONV, but no statistically significant difference in pain scores at 24 h. Dagher et al. [33] reported lower postoperative morphine consumption and higher patient satisfaction in an opioid‐sparing protocol, with no difference in LOS. Mieszczanski et al. [19] highlighted that the existing evidence base relies on heterogeneous protocols that limit definitive conclusions regarding the superiority of OFA over well‐designed opioid‐sparing regimens.

Silva et al. [40] found that a multimodal regimen combining lidocaine, dexmedetomidine, magnesium, and methadone was associated with substantially lower rates of moderate‐to‐severe PACU pain and reduced PONV compared with earlier opioid‐based institutional protocols, although increased intraoperative hypotension was observed. However, this study reflected retrospective comparison of evolving multimodal institutional protocols rather than a randomized head‐to‐head OFA trial. In contrast, the prospective randomized controlled trial by Perez et al. [21] evaluating a fully opioid‐free regimen found no significant reductions in postoperative opioid consumption, pain scores, PONV, or hospital length of stay compared with conventional anesthesia, representing an important null finding that constrains overly optimistic interpretations of OFA. Importantly, both groups in Perez et al. received substantial background multimodal analgesia and antiemetic prophylaxis, potentially attenuating detectable differences between study arms. Together, these studies suggest that OFA outcomes may depend heavily on comparator protocol intensity, background multimodal analgesia, and study design.

### 3.5. ERAS Pathways in Bariatric Surgery

#### 3.5.1. Conceptual Framework

ERAS programs are not pharmacologic interventions but structured, multidisciplinary perioperative care pathways that standardize the perioperative experience across multiple domains: preoperative patient education and carbohydrate loading; reduced fasting intervals; hemodynamic optimization; multimodal PONV prophylaxis; multimodal analgesia; goal‐directed fluid therapy; early initiation of oral intake; early mobilization; and standardized discharge criteria with structured follow‐up. The analgesic benefit of individual pharmacologic agents is not independent of the ERAS background: ERAS adherence is not merely a covariate but a potential effect modifier for pharmacologic intervention trials. The distinction between formal ERAS protocol adoption and actual high‐adherence implementation is clinically important: Centers that nominally introduce ERAS protocols but implement only a minority of recommended components may observe limited benefit, masking the true potential of the pathway.

#### 3.5.2. Evidence Summary

Table 4 summarizes the key ERAS studies. Among contemporary ERAS investigations, the multicenter POWER‐3 study by Ripollés‐Melchor et al. represents one of the largest prospective evaluations of ERAS implementation in bariatric surgery, including 2082 patients across multiple centers. Although self‐designated ERAS versus non‐ERAS pathways were not independently associated with significant differences in overall complications, readmissions, re‐interventions, mortality, or hospital length of stay, adherence analysis demonstrated that patients in the highest ERAS adherence quartile (> 72.2% adherence) experienced significantly shorter hospital stay compared with those in the lowest adherence quartile (< 55% adherence) (2 [IQR 2–3] vs. 3 [IQR 2–4] days; OR 1.54, 95% CI 1.09–2.17; *p* = 0.015), without increased postoperative complications or readmissions. These findings reinforce the concept that ERAS effectiveness in bariatric surgery may depend less on nominal pathway designation than on the degree of protocol adherence achieved in clinical practice. [22],

**TABLE 4:** Key studies evaluating ERAS pathways in laparoscopic bariatric surgery.

Study	Design	*N*	ERAS components	Adherence measured	Comparator	LOS effect	Complications	PONV	Methodological appraisal/key finding
Ripollés‐Melchor et al. [22] (POWER‐3)	Multicenter observational	2082	ERAS Society bariatric recommendations	Yes (dose–response)	Pre‐ERAS practice	Dose–response LOS reduction with adherence	No increase	Not primary	ROBINS‐I: Moderate risk. Largest single study; clearest adherence‐outcome evidence
Doshi et al. [23]	Implementation study	1804	Full ERAS program	Yes	Pre‐ERAS practice	Significantly reduced	Reduced	Reduced	ROBINS‐I: Moderate risk. Second‐largest; includes direct cost reduction evidence
Taylor et al. [24]	Implementation study	433	ERAS pathway	Partial	Pre‐ERAS practice	Reduced	Not reported	Reduced	ROBINS‐I: Moderate risk
Zhou et al. [25]	Retrospective cohort	456	ERAS vs conventional	No	Conventional care	Reduced	Reduced	Reduced	ROBINS‐I: Moderate risk (confounding)
Trotta et al. [26]	Implementation study	200	ERAS‐aligned protocol	Partial	Pre‐ERAS practice	Majority discharged ≤ 2 days	Not increased	Reduced	ROBINS‐I: Moderate risk. Early discharge feasibility data
Aleassa et al. [27]	Cost analysis	NS	ERAS pathway	Not stated	Conventional care	Not primary	Not primary	Not primary	ROBINS‐I: Moderate risk. Cost‐effectiveness beyond LOS reduction
Kearns et al. [28]	Implementation study	NS	ERAS national bariatric center	Partial	Pre‐ERAS practice	Reduced	Not increased	Reduced	ROBINS‐I: Moderate risk
Noel et al. [29]	Implementation study	NS	ERAS new bariatric center	Partial	Pre‐ERAS practice	Reduced	Not increased	Reduced	ROBINS‐I: Moderate risk
Katz‐Summercorn et al. [34]	Retrospective cohort	NS	ERAS setting	Yes	ERAS baseline	LOS predictors identified	Not primary	Not primary	ROBINS‐I: Moderate risk. Identifies BMI, sex, operative conversion as LOS predictors

*Note:* Methodological appraisal tools as in Table 1, PONV = postoperative nausea and vomiting.

Abbreviations: ERAS = enhanced recovery after surgery, LOS = length of stay.

Additional large‐scale implementation data were reported by Doshi et al., who compared 680 procedures performed before ERAS implementation with 1124 procedures performed after protocol adoption. ERAS implementation was associated with a one‐day reduction in median hospital length of stay (*p* = 0.001), an approximately $2000 reduction in median surgical costs, and significantly fewer unplanned 30‐day readmissions. Multivariable analysis demonstrated that ERAS implementation independently predicted shorter hospital stay (incidence rate ratio = 0.72, *p* < 0.001), lower median cost (−$2,230, *p* < 0.001), and reduced odds of unplanned readmission (OR = 0.48, *p* < 0.001). These findings further support the value of structured ERAS implementation as a system‐level perioperative optimization strategy in bariatric surgery [23]. Taylor et al. [24] reported reductions in LOS, costs, and readmissions. Zhou et al. [25] confirmed consistent reductions in complications, PONV, LOS, and costs. Trotta et al. [26] demonstrated the feasibility of early discharge within 2 days without increased risk. Aleassa et al. [27] provided evidence for cost‐effectiveness beyond LOS reduction. Kearns et al. [28] confirmed feasibility in a national bariatric center. Noel et al. [29] reported ERAS benefit in a newly established program. Katz‐Summercorn et al. [34] identified higher BMI, male sex, and operative conversion as independent predictors of delayed discharge within an ERAS setting.

Collectively, the ERAS evidence represents the most consistently favorable body of literature within this review, particularly regarding reductions in hospital length of stay, healthcare costs, and recovery efficiency. However, not all studies demonstrated uniform benefit across postoperative complications, readmissions, or mortality, and the observed effects likely depend on implementation fidelity and adherence intensity. Importantly, ERAS protocols varied substantially across studies in terms of multimodal analgesia strategies, PONV prophylaxis, fluid management, early feeding, mobilization targets, respiratory optimization, and discharge criteria, limiting direct comparison between studies and making it difficult to isolate which specific components drive the observed benefits. Nevertheless, the overall literature suggests that higher adherence to structured ERAS pathways is associated with improved perioperative recovery outcomes in bariatric surgery.

## 4. Discussion

### 4.1. Principal Findings

**FIGURE 2:**
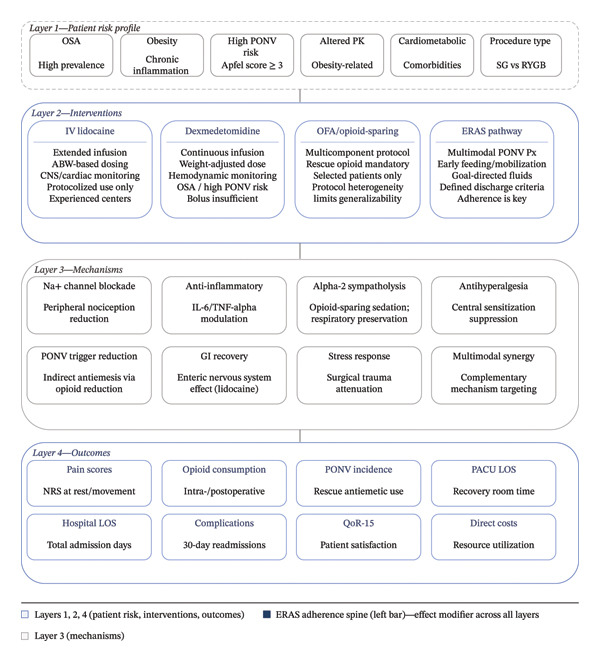
Mechanistic and implementation framework for ERAS‐guided multimodal opioid‐sparing analgesia in laparoscopic bariatric surgery. Figure description: A four‐layer conceptual model. Central node: ERAS‐guided multimodal perioperative recovery. Layer 1—Patient risk profile: Obesity (chronic inflammation, altered PK); obstructive sleep apnea; high PONV risk (Apfel score ≥ 3); cardiometabolic comorbidity; procedure type (sleeve gastrectomy vs Roux‐en‐Y gastric bypass); operative duration. Layer 2—Interventions: Intravenous lidocaine (extended protocolized infusion); dexmedetomidine (continuous infusion); opioid‐free/opioid‐sparing protocols; multimodal PONV prophylaxis; regional anesthesia/TAP blocks (acknowledged adjunct); early oral intake; early mobilization; defined discharge criteria. Layer 3—Mechanisms: Opioid consumption reduction; anti‐inflammatory cytokine modulation (IL‐6, TNF‐alpha); antihyperalgesia (dorsal horn suppression); sympatholysis and stress response attenuation; respiratory preservation; PONV trigger reduction; gut motility recovery. Layer 4—Outcomes: Postoperative pain scores; opioid consumption; PONV incidence; PACU LOS; hospital LOS; complications; readmissions; QoR‐15/patient satisfaction; direct costs. Connecting element: ERAS adherence is shown as an effect modifier linking all interventions to outcomes. Pharmacologic agents are shown as components within, not substitutes for, the ERAS framework. Abbreviations: ABW = adjusted body weight; GI = gastrointestinal; LOS = length of stay; NRS = numeric rating scale; OSA = obstructive sleep apnea; OFA = opioid‐free anesthesia; PACU = postanesthesia care unit; PK = pharmacokinetics; PONV = postoperative nausea and vomiting; Px = prophylaxis; QoR‐15 = Quality of Recovery‐15; RYGB = Roux‐en‐Y gastric bypass; SG = sleeve gastrectomy; TNF = tumor necrosis factor.

This updated narrative review synthesizes evidence on four mechanistically distinct but clinically interconnected strategies for perioperative optimization in laparoscopic bariatric surgery. The principal finding is that no single pharmacologic agent provides universally consistent benefit across all relevant outcomes and that the clinical value of each intervention is determined by its mechanistic fit, patient risk profile, dosing strategy, and integration within a structured ERAS framework. ERAS pathways provide the most consistent and reproducible improvement in perioperative outcomes, with benefit proportional to adherence. Dexmedetomidine shows the most consistent evidence for PONV reduction and perioperative opioid‐sparing when administered as a continuous infusion, but its benefit for pain scores is variable and hemodynamic monitoring is required. Intravenous lidocaine may provide analgesic, anti‐inflammatory, and antiemetic effects, but these benefits appear most plausible with extended infusion beyond the intraoperative period, adjusted body‐weight dosing, and appropriate toxicity monitoring. OFA reduces PONV and opioid consumption but does not consistently demonstrate superiority over well‐designed opioid‐sparing multimodal regimens for pain control or LOS. Together, these findings support a central thesis: In laparoscopic bariatric surgery, opioid‐sparing pharmacologic strategies are clinically meaningful only when they are mechanism‐based, patient‐selected, safety‐monitored, and embedded within high‐adherence ERAS pathways (Figure 2).

### 4.2. Mechanistic Interpretation of Heterogeneity

The heterogeneity of results across studies in each domain is not merely a methodological problem but reflects genuine mechanistic complexity. For lidocaine, the key source of clinical variability is infusion duration: The anti‐inflammatory and antihyperalgesic mechanisms that most plausibly explain recovery benefit require sustained plasma concentrations, which are not achieved with short intraoperative infusions. For dexmedetomidine, the distinction between bolus and continuous infusion explains much of the variability in pain outcomes; continuous infusion maintains alpha‐2 receptor occupancy throughout the surgical and early recovery period, while bolus dosing achieves transient peak concentrations insufficient for sustained opioid‐sparing. For OFA, the heterogeneity in protocol composition means that comparing OFA trials directly is methodologically inappropriate, since each protocol essentially constitutes a different intervention. For ERAS, the degree of adherence is the dominant source of variability: Studies measuring adherence consistently find dose–response relationships, while those without adherence reporting cannot be meaningfully compared. Recognizing these mechanistic sources of heterogeneity is essential to avoid the erroneous conclusion that negative trials imply inefficacy of the underlying pharmacologic concept (Table 5).

**TABLE 5:** Mechanistic rationale and clinical interpretation of perioperative analgesic strategies.

Strategy	Mechanistic rationale	Most supported outcome	Main uncertainty	Safety concern	Best implementation context	Evidence strength
Intravenous lidocaine	Voltage‐gated Na + channel blockade; anti‐inflammatory cytokine modulation (IL‐6, TNF‐alpha); antihyperalgesia via dorsal horn suppression; enteric recovery benefit with extended infusion	PONV reduction and opioid‐sparing with extended (24 h) infusion; IL‐6 reduction	Analgesic benefit with intraoperative‐only use; optimal dose and duration; effect on LOS	CNS/cardiovascular toxicity; altered PK in obesity; accumulation with extended infusion	Extended protocolized infusion; ABW‐based dosing; experienced center with monitoring capacity	Moderate: Meta‐analyses support benefit; null RCTs with short infusion limit universality
Dexmedetomidine	Alpha‐2 adrenergic agonism; sympatholysis; dorsal horn inhibition (spinal alpha‐2); indirect antiemesis via opioid reduction; respiratory‐sparing sedation (alpha‐2:alpha‐1 = 1600:1)	PONV reduction; perioperative opioid consumption reduction (most consistent across meta‐analyses)	Postoperative pain scores; analgesic benefit with single bolus	Bradycardia; hypotension; hemodynamic instability in low‐reserve patients; bolus‐related peaks	Continuous intraoperative infusion; weight‐adjusted dosing; hemodynamic monitoring; patients with OSA or high PONV risk	Moderate: Consistent meta‐analyses for PONV/opioid; heterogeneous pain results; null bolus trial important
Opioid‐free/opioid‐sparing anesthesia	Multicomponent opioid minimization targeting multiple pathways; elimination of opioid‐mediated PONV triggers; protocol‐specific pharmacologic rationale	PONV reduction; opioid consumption reduction (driven by antiemetic component mechanisms)	Postoperative pain control; LOS reduction; superiority over opioid‐sparing; protocol‐specific generalizability	Protocol complexity; rescue opioid requirements; risk of inadequate analgesia if applied dogmatically	Carefully selected patients (severe OSA, extreme opioid sensitivity); experienced centers; rescue opioid protocol mandatory	Moderate: Largest meta‐analysis positive for PONV/opioids; multiple null trials for pain/LOS; protocol heterogeneity limits pooling
ERAS pathways	Not pharmacologic; multidisciplinary implementation framework; adherence‐dependent synergistic multicomponent effect; ERAS adherence functions as effect modifier for pharmacologic interventions	LOS reduction (dose–response with adherence); complication reduction; PONV reduction; direct cost savings	Component‐specific effect attribution; outcomes in low‐adherence or resource‐limited settings	No pharmacologic safety concern; risk of premature discharge if discharge criteria not rigorously applied	All patients undergoing laparoscopic bariatric surgery; benefit proportional to adherence level; requires multidisciplinary commitment	High: Most consistent and largest evidence base across diverse healthcare settings; multicenter dose–response data

*Note:* AV = atrioventricular, PONV = postoperative nausea and vomiting.

Abbreviations: BMI = body mass index, bpm = beats per minute, CNS = central nervous system, ERAS = enhanced recovery after surgery, OSA = obstructive sleep apnea, PACU = postanesthesia care unit, TBW = total body weight.

### 4.3. Intravenous Lidocaine: Dose, Duration, and Patient Selection

The pattern of results across lidocaine trials is best explained by the interaction between infusion duration and the relevant mechanistic pathway. Meta‐analytic evidence supports a beneficial effect on pain, opioid consumption, and PONV when intraoperative lidocaine is pooled across trial designs [9, 10]. However, when trials are considered individually by infusion duration, those limiting administration to the intraoperative period consistently fail to demonstrate analgesic benefit [35, 36], while those extending infusion to 24 h demonstrate reductions in pain and inflammatory markers [37]. The distinction is mechanistically coherent: The antihyperalgesic effect of lidocaine requires sufficient time to suppress the development of spinal sensitization, and the anti‐inflammatory effect on IL‐6 and related cytokines is likely to become clinically relevant only with sustained systemic exposure.

Although the available evidence remains insufficient to define a universally optimal regimen, the current literature suggests that lidocaine infusions extending into the postoperative period, rather than intraoperative‐only administration, are more likely to achieve clinically meaningful recovery benefits.

Obesity‐related pharmacokinetic alterations introduce a second layer of complexity. Increased volume of distribution, altered protein binding, and modified hepatic clearance mean that standard weight‐based dosing protocols may not be directly applicable [11, 12]. Adjusted body‐weight dosing has been recommended to avoid subtherapeutic or toxic concentrations, but specific validated protocols for the bariatric population remain limited. The PROSPECT guideline for laparoscopic sleeve gastrectomy [7] does not provide a strong universal recommendation for lidocaine, reflecting the heterogeneity of the evidence and reinforcing the need for selective, protocolized use. The apparent discrepancy between favorable pooled meta‐analytic estimates and more cautious procedure‐specific recommendations highlights the need to distinguish statistical benefit under ideal trial conditions from routine clinical recommendation in heterogeneous real‐world populations.

### 4.4. Dexmedetomidine: Opioid‐Sparing and Antiemetic Effects Versus Hemodynamic Risk

Dexmedetomidine demonstrates the most consistent pharmacological rationale among the systemic agents reviewed. The mechanistic basis for opioid‐sparing through alpha‐2‐mediated sympatholysis and dorsal horn inhibition, and for PONV reduction through decreased perioperative opioid exposure, is well‐supported by the pharmacological evidence and is reflected in the most consistent findings across meta‐analyses [13, 14, 31]. The repeated null finding for pain outcomes [16, 17] is not paradoxical in this mechanistic context: The primary analgesic mechanism of dexmedetomidine operates through opioid‐sparing rather than direct nociceptive suppression, meaning that conventional numeric pain rating scales may not capture its functional benefit.

The superiority of continuous infusion over single bolus is mechanistically predictable and clinically confirmed by the contrast between null bolus studies [17] and positive infusion trials [13–16]. Continuous infusion protocols require anesthetic team familiarity, dose titration, and intraoperative hemodynamic monitoring. Nair et al. [32] confirmed that bradycardia and hypotension are real and clinically relevant concerns. The PROSPECT guideline [7] advises caution in routine adoption, reflecting the same evidence‐recommendation gap identified for lidocaine. The most clinically acceptable position is to reserve dexmedetomidine for patients in whom opioid‐sparing is particularly important: those with significant OSA, elevated Apfel PONV risk score, or prior opioid sensitivity, in centers with appropriate monitoring protocols.

### 4.5. OFA: Promising But Not Universally Superior

The most important conceptual insight from the OFA evidence is that the benefit of opioid‐free protocols appears to be driven primarily by the specific pharmacologic components used rather than by the absence of opioids per se. The reduction in PONV and opioid consumption consistently observed across OFA meta‐analyses [18, 20] is most parsimoniously explained by the pro‐antiemetic and opioid‐sparing properties of the agents constituting those protocols (dexmedetomidine, lidocaine, ketamine, and dexamethasone), rather than by opioid elimination itself. This interpretation is supported by the null finding of Perez et al. [21] with a formally opioid‐free protocol, which demonstrates that opioid elimination does not guarantee superior outcomes.

The concept of OFA is theoretically strongest for patients at highest risk of opioid‐related harm: those with severe OSA, prior respiratory events under opioid analgesia, or extreme obesity with severely compromised respiratory mechanics. However, the generalizability of OFA protocols developed in specialized centers to routine practice is uncertain. Rescue opioid availability and a prespecified threshold for opioid escalation must remain part of any OFA protocol [18, 19]. Opioid‐sparing multimodal care may represent a more pragmatic and broadly implementable perioperative strategy across heterogeneous bariatric practice settings.

### 4.6. ERAS Pathways: Implementation, Adherence, and Scalability

ERAS represents a fundamentally different class of intervention: It is an implementation framework rather than a drug, and its effects emerge from the synergistic interaction of multiple standardized components. The dose–response relationship between adherence and LOS demonstrated in POWER‐3 [22] is the clearest evidence in this review that the mechanism of ERAS benefit is adherence‐dependent and multicomponent. Pharmacologic adjuncts, including lidocaine, dexmedetomidine, and OFA protocols, are most meaningfully evaluated and implemented as components within this framework, because the effect of any single drug may be substantially modified by whether other ERAS components are simultaneously implemented.

The economic evidence from Doshi et al. [23] and Aleassa et al. [27] reinforces that ERAS generates value beyond LOS reduction alone, including reductions in complication management costs and readmission‐related resource utilization. In resource‐limited settings, high‐adherence ERAS without expensive pharmacologic adjuncts may outperform low‐adherence ERAS augmented with costly agents. The identification by Katz‐Summercorn et al. [34] of patient‐level predictors of delayed discharge underscores that even within ERAS programs, individualized risk assessment remains necessary. The clinically important distinction between PACU LOS and hospital LOS is also relevant here: Pharmacologic interventions that reduce PACU time may not translate to shorter total hospitalization without concurrent implementation of the early feeding, mobilization, and discharge readiness components of ERAS [7, 41].

### 4.7. Reconciling Meta‐Analytic Findings With Procedure‐Specific Recommendations

A recurring tension in this review is the apparent discrepancy between favorable pooled estimates from meta‐analyses and more cautious recommendations from procedure‐specific guidelines, such as PROSPECT [7]. Meta‐analyses aggregate results across heterogeneous trial populations, dosing strategies, and perioperative contexts to estimate an average treatment effect. Procedure‐specific guidelines apply a more conservative evidence threshold, weighting the risk of adverse effects, the relevance of the specific procedural context, and the degree of evidence certainty before issuing routine recommendations. Statistical significance in a pooled estimate does not automatically imply that the intervention should be routinely recommended across all patients and centers. The most appropriate clinical interpretation of the evidence in this review is therefore that lidocaine and dexmedetomidine may benefit carefully selected patients within protocolized programs, rather than that they should be universally adopted based on favorable meta‐analytic pooling.

### 4.8. Patient Selection and Safety Considerations

Table 6 summarizes safety and patient selection considerations for each intervention. The pharmacologic strategy most likely to optimize outcomes in any individual patient depends on the convergence of procedure type, baseline risk profile, institutional capacity, and ERAS adherence level. Several patient subgroups warrant specific consideration.

**TABLE 6:** Safety and patient selection considerations for pharmacologic adjuncts in laparoscopic bariatric surgery.

Strategy	Potential benefit	Main safety concern	Patients most likely to benefit	Patients requiring caution	Monitoring requirements	Relative contraindications	Implementation notes
Intravenous lidocaine	Analgesic; anti‐inflammatory (IL‐6 reduction); antiemetic; possible GI recovery benefit with extended infusion	CNS toxicity (perioral numbness, dizziness, seizures at high concentrations); cardiovascular toxicity (QRS widening, arrhythmias, hypotension); drug accumulation with prolonged infusion in obesity	Patients with significant postoperative inflammatory response; moderate–high PONV risk; candidates for extended infusion protocols	Patients with significant hepatic dysfunction (altered clearance); use of antiarrhythmic drugs (Class I); known sodium channel disorder; centers without infusion monitoring protocols	Continuous cardiac monitoring; periodic neurological assessment during infusion; serum level monitoring recommended for extended protocols in obesity	Significant hepatic insufficiency; concomitant Class Ia/Ib antiarrhythmics; known channelopathies; hemodynamic instability	ABW‐based dosing mandatory; do not use TBW in obesity; institutional infusion protocol required; intraoperative‐only use insufficient for consistent benefit
Dexmedetomidine	Opioid‐sparing; PONV reduction; respiratory‐sparing sedation; hemodynamic stability vs remifentanil; sympatholysis	Bradycardia (dose‐dependent); hypotension; AV conduction block; delayed emergence with excessive sedation; bolus‐related hemodynamic instability	OSA patients; high Apfel score ( ≥ 3); patients with prior opioid‐related respiratory events; prolonged surgical procedures	Preexisting bradycardia (< 50 bpm); high‐degree AV block; hemodynamically unstable patients; significant heart failure; severe volume depletion	Continuous intraoperative hemodynamic monitoring; HR and BP monitoring at minimum 5‐min intervals; vasopressor availability	High‐degree AV block (without pacemaker); preexisting clinically significant bradycardia; use of other negative chronotropic agents without cardiac backup	Continuous infusion preferred over single bolus; avoid rapid bolus loading; have atropine and vasopressor immediately available; dosing: 0.4–0.8 mcg/kg/h titrated
Opioid‐free/opioid‐sparing anesthesia	Reduced perioperative opioid exposure; PONV reduction; potential respiratory safety benefit in OSA	Inadequate analgesia if applied dogmatically; hemodynamic instability from multicomponent protocols; complexity‐related adverse events (ketamine dysphoria, dexmedetomidine bradycardia, lidocaine toxicity); delayed identification of inadequate analgesia	Severe OSA with documented opioid sensitivity; extreme obesity (BMI > 60) with severely compromised respiratory mechanics; prior adverse respiratory events under opioid analgesia; high Apfel PONV risk	Patients with hemodynamic instability or cardiac comorbidity limiting dexmedetomidine; hepatic dysfunction limiting lidocaine; low‐resource centers without multicomponent monitoring capability	Component‐specific monitoring as above; PACU enhanced monitoring for first 4 h; mandatory pain reassessment protocol; rescue opioid threshold prespecified	Inability to monitor multiple agent‐specific adverse effects simultaneously; unreliable IV access for rescue; absence of anesthesiologist experienced with multicomponent protocols	Prespecify rescue opioid threshold and escalation protocol; educate PACU staff on multicomponent adverse event profiles; not appropriate for all bariatric patients; opioid‐sparing may be safer universal standard
ERAS pathways	LOS reduction proportional to adherence; complication reduction; PONV reduction; cost savings; early discharge feasibility; patient satisfaction improvement	Premature discharge if discharge criteria applied inconsistently; readmission risk if patient education inadequate; risk of complications not identified before discharge	All patients undergoing laparoscopic bariatric surgery who meet preoperative optimization criteria	Patients with multiple complex comorbidities requiring longer monitoring; patients in low‐resource settings where ERAS components cannot be fully implemented; patients with discharge barriers (social, geographic)	Component‐specific monitoring per individual ERAS elements; standardized discharge checklist; 30‐day readmission and complication tracking	No absolute contraindications to ERAS framework; individual components may be contraindicated in specific patients	Adherence measurement is essential, not optional; distinguish protocol adoption from high‐adherence implementation; pharmacologic adjuncts function as ERAS components, not substitutes for the full pathway

*Note:* GI = gastrointestinal, IL‐6 = interleukin‐6, PONV = postoperative nausea and vomiting, PK = pharmacokinetic.

Abbreviations: ABW = adjusted body weight, CNS = central nervous system, LOS = length of stay, OFA = opioid‐free anesthesia, OSA = obstructive sleep apnea, PACU = postanesthesia care unit, TNF‐alpha = tumor necrosis factor‐alpha.

Patients with significant OSA represent the highest‐priority group for opioid‐minimizing strategies: Opioid‐induced respiratory depression in this population may be life‐threatening, particularly in the early recovery period before full emergence from residual anesthetic effects. Dexmedetomidine and OFA protocols that substantially reduce intraoperative opioid exposure are mechanistically well‐justified for this subgroup. The high Apfel PONV risk score, commonly encountered in bariatric cohorts (female sex, nonsmoker, history of PONV or motion sickness, and opioid use), identifies patients in whom dexmedetomidine, lidocaine extended infusion, and multimodal antiemetic prophylaxis should be prioritized. Procedure type modifies the analgesic environment: Sleeve gastrectomy and Roux‐en‐Y gastric bypass differ in operative time, level of visceral dissection, and anatomical complexity, and pharmacologic protocols may need to be tailored accordingly. Longer operative duration may favor continuous dexmedetomidine infusion over bolus and extended lidocaine infusion over intraoperative‐only administration. Patients with cardiometabolic comorbidity, including conduction abnormalities, baseline bradycardia, or significant heart failure, require particular caution with dexmedetomidine. For intravenous lidocaine, hepatic dysfunction and use of antiarrhythmic drugs represent relative contraindications. Institutional resources and monitoring capacity must also be considered: Extended lidocaine infusions and continuous dexmedetomidine protocols require nursing monitoring protocols and institutional experience that may not be uniformly available.

### 4.9. Clinical Implications

For clinical practice, the evidence most consistently supports a structured ERAS program incorporating multimodal, opioid‐sparing analgesia as the primary perioperative strategy. Within this framework, dexmedetomidine as a continuous intraoperative infusion is a reasonable adjunct in patients with OSA, elevated PONV risk, or significant opioid sensitivity, administered with hemodynamic monitoring and weight‐adjusted dosing. Intravenous lidocaine may offer anti‐inflammatory and antiemetic benefit with extended protocolized infusion in experienced centers; intraoperative‐only administration appears insufficient to achieve consistent analgesic benefit. OFA protocols may be appropriate in highly selected patients in experienced centers, but should not be routinely adopted without rescue opioid protocols and individualized analgesic assessment. In all cases, these pharmacologic strategies are adjuncts to structured ERAS implementation, not replacements for it.

The clinically important distinction between PACU LOS and hospital LOS [41] should inform how outcomes are measured and reported. An intervention may meaningfully reduce PACU time through faster emergence and lower PONV without affecting total hospital stay if broader ERAS components are not concurrently implemented. Sex and gender differences in pain perception, opioid metabolism, PONV susceptibility, and pharmacodynamic responses to dexmedetomidine and lidocaine deserve specific attention in future trials and institutional protocols.

### 4.10. Limitations

Several limitations of this review should be acknowledged. The narrative design introduces potential selection and interpretation bias despite systematic search and dual‐reviewer extraction procedures. Substantial heterogeneity exists across included studies in dosing regimens, comparators, procedure types, outcome definitions, and background ERAS adherence, limiting the ability to draw definitive conclusions about individual interventions. Gray literature and trial registries were not systematically searched, which may have introduced publication bias toward positive findings. Earlier versions of the review were based on a more limited database search strategy, although the final revised review incorporated PubMed/MEDLINE, Embase, Scopus, and the Cochrane Library to improve comprehensiveness and reduce selection bias. The methodological appraisal, while structured using RoB 2.0, ROBINS‐I, and AMSTAR‐2, was applied in the context of a narrative review and does not constitute a formal systematic appraisal equivalent to a Cochrane‐level review. ERAS adherence was not uniformly reported across pharmacologic intervention studies, representing a significant uncontrolled source of variability. Finally, new references incorporated from 2024 to 2026 include some papers available only as ePub ahead of print, and full methodological details may be subject to change upon final publication.

Therefore, the findings should be interpreted as a structured narrative synthesis intended to contextualize contemporary evidence rather than as a formal systematic review or clinical practice guideline.

### 4.11. Future Research Priorities

Future investigations should prioritize multicenter RCTs with adequate sample sizes, standardized dosing protocols, detailed ERAS adherence reporting, and patient‐centered outcome measures. For lidocaine, key research needs include the optimal infusion duration and dose, adjusted body‐weight dosing validation in the bariatric population, and whether the benefit is specific to extended infusion versus intraoperative use alone. For dexmedetomidine, comparative trials of continuous infusion versus bolus administration, optimal dosing ranges, and prospective hemodynamic monitoring protocols are needed. For OFA, head‐to‐head trials comparing opioid‐free versus opioid‐sparing regimens within standardized high‐adherence ERAS programs are essential. Outcome measures should include QoR‐15, patient satisfaction, functional recovery, PACU LOS, hospital LOS, PONV, complications, readmissions, and direct healthcare costs. Subgroup analyses by procedure type, sex and gender, OSA severity, Apfel score, operative duration, BMI category, and ERAS adherence level would substantially advance the field. Implementation science frameworks should be applied to understand the barriers and facilitators to ERAS adoption across different resource settings. Future studies should also evaluate sex‐specific pharmacodynamic responses and resource‐stratified implementation strategies to improve the generalizability and real‐world applicability of opioid‐sparing perioperative protocols in bariatric surgery.

## 5. Conclusions

ERAS‐guided multimodal opioid‐sparing perioperative care represents the most comprehensive and evidence‐supported framework for optimizing outcomes in laparoscopic bariatric surgery. The clinical value of pharmacologic adjuncts within this framework is mechanistically grounded, patient‐dependent, and proportional to the quality of ERAS implementation. Dexmedetomidine, administered as a continuous intraoperative infusion, reduces perioperative opioid exposure and PONV through alpha‐2‐mediated sympatholysis and opioid‐sparing mechanisms, but its analgesic benefit is variable and hemodynamic monitoring is required for safe use. Intravenous lidocaine may provide analgesic, anti‐inflammatory, and antiemetic effects through sodium channel blockade and cytokine modulation, but these benefits appear most consistent with extended protocolized infusion and adjusted body‐weight dosing in centers with appropriate monitoring capacity. OFA may reduce PONV and opioid exposure in selected patients, but current evidence does not consistently demonstrate superiority over well‐designed opioid‐sparing multimodal regimens for pain control or length of stay; opioid‐sparing care may be more broadly applicable. These pharmacologic strategies are best understood as selective, mechanism‐based adjuncts to be integrated within high‐adherence ERAS pathways rather than as universal standalone interventions. In laparoscopic bariatric surgery, opioid‐sparing pharmacologic strategies are clinically meaningful only when they are mechanism‐based, patient‐selected, safety‐monitored, and embedded within structured perioperative recovery programs. Rigorous multicenter randomized trials comparing opioid‐free versus protocolized opioid‐sparing ERAS pathways, with standardized and yet personalized dosing regimens, detailed ERAS adherence reporting, procedure‐specific subgroup analyses, and patient‐centered outcomes, such as quality of recovery, functional recovery, respiratory complications, and persistent opioid use, are required to refine optimal implementation strategies in bariatric surgery populations, particularly among patients with OSA and elevated PONV risk.

## Author Contributions

Carlos Zavaleta‐Corvera: conceptualization, methodology, literature review, data curation, formal analysis, writing–original draft, and writing–review and editing. Erick Auccacusi Pachari, Wendy Farfán‐Martinez, Julian Espinoza‐Portilla, and Mario Bolarte‐Arteaga: literature review, critical revision of the manuscript, and writing–review and editing.

## Funding

The authors declare that this research did not receive any specific grant from funding agencies in the public, commercial, or not‐for‐profit sectors.

## Disclosure

All authors approved the final manuscript.

## Ethics Statement

All authors certify that they meet the current authorship criteria of the International Committee of Medical Journal Editors (ICMJE).

## Conflicts of Interest

The authors declare no conflicts of interest.

## Data Availability

Data sharing is not applicable to this article as no datasets were generated or analyzed during the current study.
